# Revisiting the origin of interleukin 1 in anamniotes and sub-functionalization of interleukin 1 in amniotes

**DOI:** 10.1098/rsob.220049

**Published:** 2022-08-17

**Authors:** Eva Hasel de Carvalho, Eva Bartok, Helen Stölting, Baubak Bajoghli, Maria Leptin

**Affiliations:** ^1^ European Molecular Biology Laboratory (EMBL), Directors' Research, Meyerhofstrasse 1, 69117 Heidelberg, Germany; ^2^ Institute of Clinical Chemistry and Clinical Pharmacology, University Hospital, University of Bonn, Venusberg Campus 1, 53127 Bonn, Germany; ^3^ Unit of Experimental Immunology, Institute of Tropical Medicine, 2000 Antwerp, Belgium; ^4^ National Heart and Lung Institute, Faculty of Medicine, Imperial College London, London, UK; ^5^ Department of Hematology, Oncology, Immunology, and Rheumatology, University Hospital of Tübingen, Otfried-Müller-Strasse 10, 72076 Tübingen, Germany

**Keywords:** evolution, cytokine, medaka, innate immune system

## Abstract

The cytokine interleukin 1 (IL-1) is an evolutionary innovation of vertebrates. Fish and amphibian have one *IL1* gene, while mammals have two copies of *IL1*, *IL1A* and *IL1B*, with distinct expression patterns and differences in their proteolytic activation. Our current understanding of the evolutionary history of IL-1 is mainly based on phylogenetic analysis, but this approach provides no information on potentially different functions of IL-1 homologues, and it remains unclear which biological activities identified for IL-1α and IL-1β in mammals are present in lower vertebrates. Here, we use *in vitro* and *in vivo* experimental models to examine the expression patterns and cleavage of IL-1 proteins from various species. We found that IL-1 in the teleost medaka shares the transcriptional patterns of mammalian IL-1α, and its processing also resembles that of mammalian IL-1α, which is sensitive to cysteine protease inhibitors specific for the calpain and cathepsin families. By contrast, IL-1 proteins in reptiles also include biological properties of IL-1β. Therefore, we propose that the duplication of the ancestral *IL1* gene led to the segregation of expression patterns and protein processing that characterizes the two extant forms of IL-1 in mammals.

## Graphical abstract

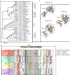

## 1. Introduction

The interleukin-1 (IL-1) family of cytokines orchestrates the immune response by mediating intercellular communication between many different cell types. Activated IL-1 has a range of inflammatory effects from fever induction to haematopoiesis and antibody synthesis (summarized in [1]). Like other immune-related cytokine genes, *IL1* genes are fast-evolving, driven by the need of the immune system to adapt to constantly changing threats.

*IL1* gene has evolved from a common ancestor approximately 420 Ma around the emergence of the gnathostomes because this gene is missing in the genomes of invertebrates and jawless vertebrates [2,3]. Only one *IL1* gene is found in the genomes of most anamniotes (fishes and amphibians), although some teleost species such as rainbow trout [4] and carp [5] have two copies, probably due to species-specific gene duplication events. The presence of *IL1A* and *IL1B* genes in all mammals and their localization on the same chromosome suggest that a tandem gene duplication event has occurred in their common ancestor [6,7].

The biological activities of IL-1α and IL-1β have been extensively analysed in mice and humans. The two cytokines share a common transduction pathway but differ in their expression patterns and activation processes [8]. At the transcriptional level, *IL1A* is constitutively expressed in a variety of cell types of haematopoietic and non-haematopoietic origin, such as keratinocytes, endothelial cells and the mucosal epithelium [9,10], whereas *IL1B* expression is predominantly induced in haematopoietic cells in response to inflammation [1]. *IL1B* is also strongly expressed in various cancer cell types [11]. The IL-1α protein is biologically active both in its full-length and cleaved forms, while the IL-1β full-length protein needs to be enzymatically cleaved to become active. The processing of the two IL-1 paralogues is regulated by distinct mechanisms. Both IL-1α and IL-1β can be processed by multiple proteases [12]. However, IL-1β is processed most efficiently by Caspase-1 [13], which, after its activation by the inflammasome [14], cleaves IL-1β at two distinct sites [15]. Caspase-1-mediated processing also results in the most bioactive form of IL-1β [12]. By contrast, Caspase-1 cannot process the IL-1α protein [15], which can instead be cleaved by Calpain proteases [16,17] and Granzyme B [18]. To what extent, the biological activities of mammalian IL-1α and IL-1β are conserved in anamniotes is not known.

Thus far, the single *IL1* gene found in the genomes of lower vertebrates has been interpreted as being most closely related to mammalian *IL1B* and is therefore seen as a functional homologue. This assumption is mainly based on phylogenetic analysis [3,19]. However, the overall low conservation of IL-1 proteins between species justifies a reassessment of this interpretation and the consideration of other characteristic factors, such as gene expression patterns and protein processing mechanisms, to support a definite assignment. Here, we compare characteristics other than peptide sequences between IL-1 proteins of anamniotes and mouse IL-1α and IL-1β. We have created a reporter for *in vivo* visualization of the expression patterns and processing of IL-1 in transgenic medaka (*Oryzias latipes*) and tested *in vitro* the dependence of cleavage of IL-1 proteins from various anamniote species on Caspase-1. Our results show that the medaka orthologue is expressed and processed in a manner similar to mammalian IL-1α and that a conserved Caspase-1 cleavage site is already present in amniotes.

## 2. Results and discussion

### 2.1. Evolution of interleukin-1 in vertebrates

**Figure 1.**
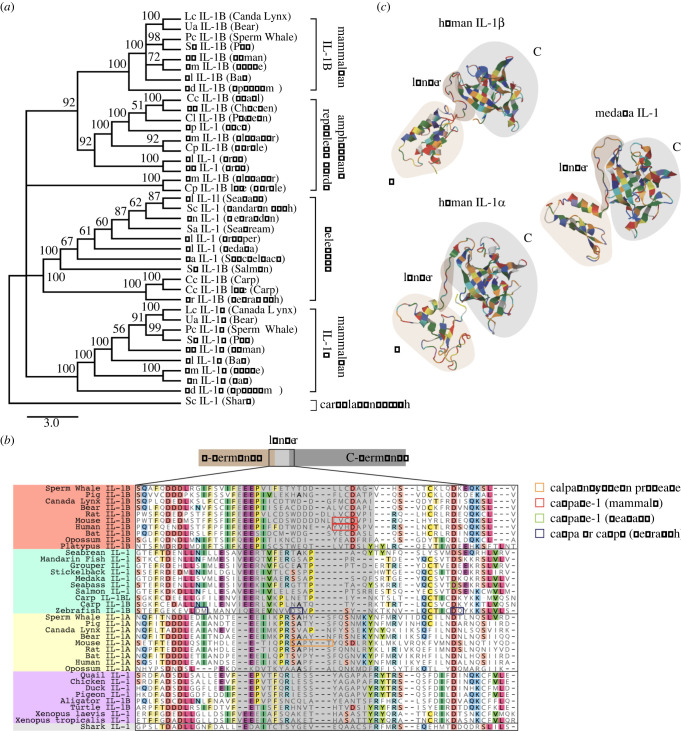
Phylogenetic analysis of IL-1 in vertebrates. (*a*) A rooted tree calculated by neighbour-joining obtained from a Clustal W alignment of IL-1 full-length proteins. Calculated distance values are indicated for each branch. The accession numbers of genes used in this analysis are listed in the electronic supplementary material, table S1. Abbreviations: Am, *Alligator mississippiensis*; Ap, *Anas platyrhynchos*; Cc, *Coturnix coturnix*; Cc, *Cyprinus carpio*; Cl, *Columba livia*; Cp, *Chrysemys picta bellii*; El, *Epinephelus lanceolatus*; Dr, *Danio rerio*; Ga, *Gasterosteus aculeatus*; Gg, *Gallus gallus*; Hs, *Homo sapiens*; Lc, *Lynx canadensis*; Ml, *Myotis lucifugus*; Mm, *Mus musculus*; Sc, *Siniperca chuatsi*; Sc, *Scyliorhinus canicula*; Ss, *Sus Scofa*; Ss, *Salmo salar*; Oa, *Ornithorhynchus anatinus*; Ol, *Oryzias latipes*; Pc, *Physeter catodon*; Ua, *Ursus americanus*; Xl, *Xenopus laevis*; Xt, *Xenopus tropicalis*. (*b*) An alignment of full-length IL-1 amino acid sequences from 28 species, showing the linker region (brown; corresponding to amino acids of linker as predicted by three-dimensional structure of human IL-1β) and surrounding sequences. Experimentally confirmed IL-1 cleavage sites are marked with boxes as indicated. (*c*) Three-dimensional structures of medaka IL-1 compared to human IL-1α and IL-1β as predicted by RaptorX.

Comparing nucleotide or amino acid sequences between species is a common method to elucidate evolutionary relationships. However, the comparison of fast-evolving genes across longer evolutionary times can be difficult, especially if pressures to diversify are active. In a phylogenetic tree of IL-1 proteins from lower and higher vertebrates, teleost IL-1 proteins form a separate cluster and share a branch point with clusters for mammalian IL-1α and IL-1β (figure 1*a*), indicating that the amino acid sequences of IL-1α and IL-1β are equally distant from teleost IL-1, mostly consistent with what has been shown by other studies [2,3,19,20]. This is also true for avian and amphibian IL-1 proteins, which together form a separate cluster. Therefore, an accurate assignment of IL-1 proteins in lower vertebrates as homologues to either IL-1α or IL-1β on the basis of phylogenetic analysis is not possible. Another criterion that can help assign ancestral relationships of genes is the comparison of their genomic localization, i.e. synteny of neighbouring genes across longer genomic stretches. The regions of vertebrate genomes in which the *IL1* genes are located are overall highly conserved, but this provides no helpful information because mammalian IL-1α and IL-1β are located next to each other within the same synteny group due to a tandem duplication event [3]. We therefore examined the conservation of characteristic amino acid sequences for IL-1α or IL-1β proteins that are relevant for their proteolytic processing. Alignment of IL-1 proteins from mammals, amphibians, reptiles, birds, teleosts and cartilaginous fishes showed that known cleavage sites in mammalian IL-1α and IL-1β are poorly conserved in lower vertebrates (figure 1*b*). Although all IL-1 proteins have the same structure in which the N- and the C-terminal domains are separated by a linker that contains potential cleavage sites (figure 1*c*), many lower vertebrates lack the conserved aspartic acid residue as a Caspase-1 cleavage sites in this linker as well as the conserved β-trefoil fold that is characteristic for mammalian IL-1β (figure 1*b*). Previous studies showed that the zebrafish IL-1 protein can be cleaved by Caspase A and Caspase B in transfected HEK cells [21–23]. However, only one of the three aspartic acid residues identified as potential substrates of Caspase-1 homologues (Caspa and Caspb) in zebrafish IL-1 [19,23] can be cleaved by Caspase-1 in the sea bass [24]. This aspartic acid residue in zebrafish IL-1 can be found in avian IL-1 but not in mammalian IL-1β. Besides the Caspase-1 site, the potential cleavage sites for other proteases such as Calpains, Cathepsins or Elastase are poorly conserved in IL-1 homologues (figure 1*b*). Therefore, protein alignments are not sufficient to assign IL-1 genes in lower vertebrates as direct ancestors of either IL-1α or IL-1β in mammals or even to deduce the function of an IL-1 common ancestor. Also, to what extent the biological activity of IL-1 proteins in anamniotes depends on their processing is still unknown.

### 2.2. Expression of medaka *il1* in naive and upon infection or local injury

**Figure 2.**
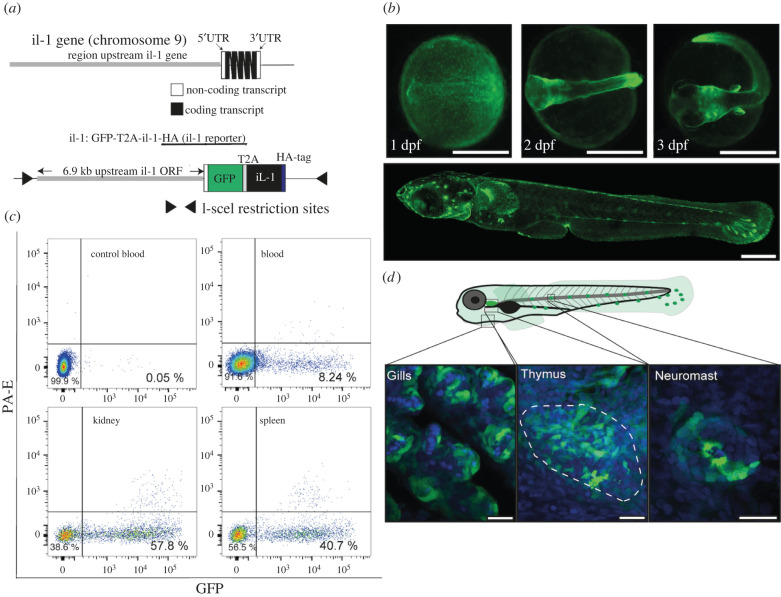
An *in vivo* reporter for medaka IL-1. (*a*) Top, schematic of the *il1* locus on medaka chromosome 9. Bottom, the transgenic *il1* reporter construct indicating the position of 6.9 kb genomic fragments upstream of the *il1* gene that drives GFP and medaka full-length *il1* cDNA with a C-terminal HA tag. (*b*) GFP expression in the transgenic *il1* reporter during embryogenesis. (*c*) Flow cytometry of haematopoietic cells isolated from blood, kidney and spleen of naive adult *il1* reporter fish. Blood from non-transgenic fish was used as a control. Data are representative of two independent biological samples. (*d*) GFP expression in the epithelial compartment of the gills, thymus and neuromast in the transgenic *il1* reporter larvae. Nuclei are stained with DAPI (blue). Scale bars in (*b*) and (*d*) indicate 500 and 20 µm, respectively.

To better understand the evolutionary history of IL-1, we performed a comprehensive comparative analysis. One aspect that distinguishes mammalian IL-1α and IL-1β is their distinct expression profiles. *IL1A* is constitutively expressed at high level in various cell types, including epithelial and haematopoietic cells, while *IL1B* expression is weak but strongly inducible in monocytic cells in response to inflammation [1,25]. To determine the expression activity of the *il1* gene in lower vertebrates, we use medaka as a model. We performed whole-mount *in situ* hybridization (WISH) with a probe for the *il1* full-length transcript but could not detect expression in naive embryos. However, in embryos injected with *E. coli*, *il1* was strongly expressed (electronic supplementary material, figure S1), which is consistent with previous observations in zebrafish [26]. Because it was not clear whether the absence of *il1* staining in naive embryos is due to insufficient sensitivity of the WISH, we created an *il1* transgenic reporter fish, in which a 6.9 kb long *il1* promoter drives the transcription of a t2a-based bi-cistronic mRNA [27] encoding GFP and medaka full-length *il1* tagged with a haemagglutinin (HA) peptide at the C-terminus (figure 2*a*). This reporter allowed us not only to reveal the spatial expression patterns of *il1* gene but also to assess the processing of the IL-1 protein under various conditions using an HA-specific antibody. The GFP signal was detectable as a weak fluorescence signal in the epidermis of live embryos at 1 day post-fertilization (dpf) (figure 2*b*; electronic supplementary material, figure S2). At later stages, the GFP signal became restricted to the epithelial compartment of the skin, gills and thymus as well as the neuromasts of the lateral line (figure 2*c*). Similar to our observation, zebrafish *il1* is expressed in the skin, gills and thymus [28,29]. Furthermore, human *IL1A* is expressed in keratinocytes and thymic epithelial cells [30]. These findings suggest that *il1* expression in the epithelial compartment, which is characteristic for mammalian *IL1A*, is conserved among vertebrates.

We also analysed the *il1* expression in the adult haematopoietic cells and performed flow cytometry of isolated cells from blood, kidney and spleen (figure 2*c*). As negative control, blood of non-transgenic fish was used. Eight per cent of blood cells, 57.8% of kidney cells, and 40.7% of spleen cells were GFP-positive. The constitutive expression of medaka *il1* in haematopoietic cells is consistent with zebrafish *il1* [28,29] and mouse *IL1a* [31]. This result further supports the notion that regulatory elements controlling the constitutive expression of *IL1A* are also conserved in lower vertebrates.

**Figure 3.**
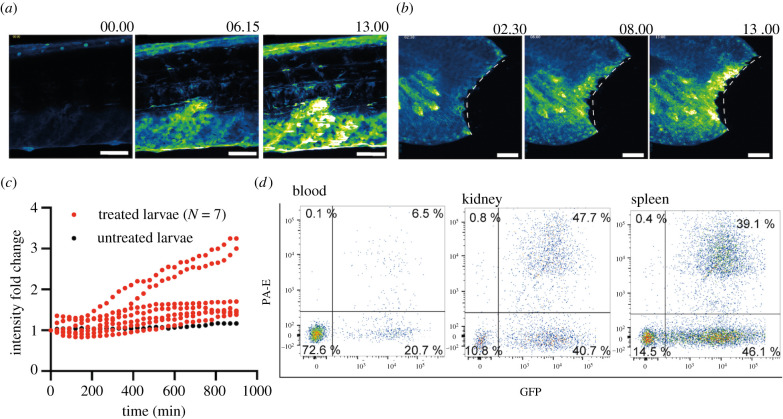
Induction of medaka *il1* upon injury and infection. (*a*) Still photographs from a time-lapse recording illustrating GFP upregulation in response to injection of 50 µM LPS into the muscle tissue of a transgenic *il1* reporter larva. Numbers indicate time in hours. (*b*) Still photographs from a time-lapse recording illustrating GFP upregulation in response to a tail-fin cut of a transgenic *il1* reporter larva. The dashed lines indicate the cut site. Numbers indicate time in hours. Scale bars in (*a*) and (*b*) indicate 50 µm. (*c*) The fold-change of mean GFP intensity quantified in the tail fin upon injury compared to untreated larvae. (*d*) Flow cytometry of haematopoietic cells isolated from blood, kidney and spleen of il1 reporter fish 16 h after subcutaneous injection of bacterial debris conjugated with Alexa 594. The data from figure 2*c* and 3*d* come from the same experiment, and the untreated group shown in figure 2c is therefore the control for this panel. Data are representative of two independent experiments.

Besides their constitutive expression, *il1* genes in lower vertebrates are inducible by inflammatory stimuli [28,29]. Our WISH analysis further confirms this observation (electronic supplementary material, figure S1). To distinguish whether *il1* inducibility is restricted to epithelial compartments or haematopoietic cells, we performed local injury and subcutaneous injection of bacteria in the transgenic fish. The GFP signal increased substantially in the epidermis when 50 µM lipopolysaccharide (LPS) was injected into the muscle tissue (figure 3*a*) or when the tail fins of transgenic larvae were injured (figure 3*b,c*) indicating that *il1* expression can be induced in non-haematopoietic cells. Next, we subcutaneously injected bacterial debris conjugated with Alexa Fluor 594 into adult transgenic fish and analysed haematopoietic cells, isolated from blood, kidney and spleen using flow cytometry 16 h-post-injection. We identified cells that expressed *il1* and had engulfed bioparticles by their combined red and green fluorescence (6.5%, 47.7% and 39.1% GFP^+^/RFP^+^ cells in blood, kidney and spleen, respectively; data from two independent experiments). The presence of GFP^+^/RFP^+^ cells in the blood and spleen (figure 3*d*; electronic supplementary material, figure S3) indicated that that all myeloid cells that had engulfed bioparticles also expressed the *il1* gene. Whether *il1* expression was induced in them locally and they then migrated into the spleen, as a secondary lymphoid organ, to initiate the adaptive immune response cannot be deduced from this data. Taken together, our results reveal that *il1* is constitutively expressed in various epithelial tissues and can be upregulated in keratinocytes and myeloid cells upon infection or local injury. Therefore, the expression pattern of medaka *il1* resembles that of mammalian *IL1A* which is both constitutive and inducible [32–34].

### 2.3. Processing of medaka interleukin-1 by proteases *in vivo*

**Figure 4.**
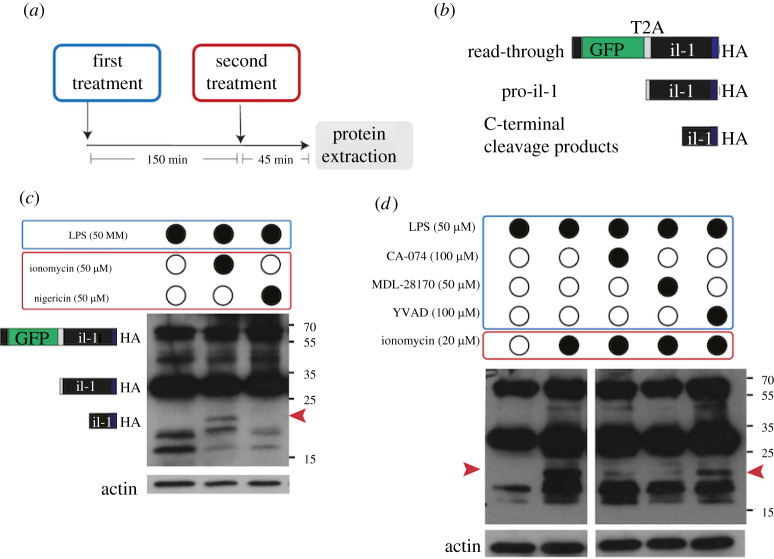
*In vivo* cleavage of medaka Il-1 upon chemical stimulation. (*a*) The experimental rationale. Freshly hatched medaka larvae were first treated with LPS for 150 min and then with a second compound (either nigericin or ionomycin) for an additional 30–45 min before protein extraction. (*b*) Schematic description of three predicted protein products that can be detected by HA antibody on WB. A fusion protein of GFP-t2a-il1-HA resulting from failure of the t2a-induced ribosome skipping has a predicted MW of 58 kDa. The size of the Il-1 pro-peptide is 29 kDa. The molecular weight of cleaved Il-1 products was predicated between 16.8 and 20.8 kDa for a cleavage site located within the linker region. (*c*,*d*) WB analysis from medaka larvae after treatment with chemical compounds using HA, GFP and actin antibodies. Red arrowheads indicate the processed Il-1 protein. In (*d*), il1 reporter larvae were additionally treated with either cysteine protease inhibitors (MDL-28170 and CA-074) or caspase-1 inhibitor Y-VAD. Note the appearance of an Il-1 cleaved form only after LPS and Ionomycin treatment (*c*), which was reduced after MDL-28170 or CA-074, but not YVAD treatment (*d*). Data are representative of four independent experiments. Additional controls for protein lysates of untreated fish and different ionomycin exposure conditions can be found in the electronic supplementary material, figure S4.

To investigate the processing of medaka IL-1 in response to inflammatory stimuli, we used an anti-HA antibody to detect the transgenic, C-terminally tagged IL-1 in whole-fish lysates on Western blots (WB). To prime immune cells, freshly hatched transgenic larvae were first exposed to LPS for 150 min (figure 4*a*). In a second treatment, we added either nigericin or ionomycin for an additional 45 min before lysates from whole larvae were prepared. Nigericin acts as a potassium ionophore that activates the NLRP3 inflammasome [35], which is required for the Caspase-1-dependent cleavage and secretion of mammalian IL-1β (reviewed in: [36]). By contrast, ionomycin is a membrane-permeable calcium ionophore that increases intracellular calcium levels triggering Calpain activation and mature IL-1α release [37,38]. The IL-1 pro-peptide is estimated to be around 29 kDA, and the C-terminal cleavage products are expected to be between 16.8 and 19.8 kDa if the precursor is cleaved within the linker region between the N- and C-terminal domains (the predicted products are schematically depicted in figure 4*b*). WB analysis showed several bands for IL-1 (figure 4*c*). In the control group, we detected HA-positive proteins around 29 and 58 kDa. The latter product is probably a read-through of the GFP-T2A-Il1 open reading frame that occurs when T2A-induced ribosome skipping is not complete, as is the case for around 10–20% of peptides in zebrafish [39]. We do not expect the read-through to influence the cleavage of IL-1 since the detected HA-tag is located at the C-terminus while GFP is linked to the N-terminus. An additional protein fragment with a size around 20 kDa was detected when larvae were treated with ionomycin (in this case 50 µM for 20 min or 20 µM for 45 min) (figure 4*c*,*d*). This peptide was not detected upon treatment with LPS alone or LPS with the potassium ionophore nigericin, a commonly used inflammasome activator. LPS treatment alone did not affect cleavage of IL-1 (electronic supplementary material, figure S4) and was therefore used as a control treatment.

The 20 kDa IL-1 protein was still present when larvae were treated with the Caspase-1 inhibitor Ac-YVAD-cmk. By contrast, when larvae were treated with cysteine protease inhibitors MDL-28170 and CA-074 prior to and during ionomycin treatment (20 µM), the 20 kDa protein was not detectable. The effect of ionomycin is dose, time and batch-dependent. While 50 µM affects fish health and IL-1 cleavage after approximately 20 min, a lower dose affects fish less severely but still leads to efficient IL-1 cleavage after approximately 45 min (e.g. in electronic supplementary material, figure S4). In the experiments comparing ionomycin to nigericin, which is a direct potassium ionophore, we used a high dose of 50 µm. However, for the inhibitor treatments, we used a lower dose of 20 µM of Ionomycin to avoid rapid lethality and increase exposure time to inhibitors. Together, our result indicates that medaka IL-1 can be processed by cysteine proteases from one or both of these protein families because MDL-28170 and CA-074 inhibit Calpains and proteases of the Cathepsin family [40,41].

We also assessed the spatial expression patterns of *calpains* and their small subunit *capns1* as well as *cathepsin B*, *L* and *S* in medaka embryos. WISH analysis showed that *calpain2*, *capns1b* and c*athepsin L2* and *S* are all expressed in the skin and gut, with enhanced expression in neuromasts (electronic supplementary material, figure S5). Their colocalization with *il1* expression makes them potential candidates for IL-1 processing enzymes in medaka.

Although our results show that medaka IL-1 is processed in response to increased intracellular Ca^2+^ levels, we can only speculate on the way it is released from the cell. IL-1 is a leaderless cytokine, secreted in an ER/Golgi-independent manner. Gasdermin (GSDM) pores have been shown to be important for unconventional secretion of IL-1 [42,43], and Gasdermin activation must be tightly controlled to avoid pyroptotic cell death induction [44]. Although the role of GSDM appears to be conserved [45], it remains elusive whether they play a role in IL-1 secretion in lower vertebrates. It is worth noting that teleost fishes possess a homologue of Gasdermin E (GSDME) but not GSDMD. In mammals, the latter protein is cleaved by Caspase-1 downstream of the classical inflammasome cascade and IL-1 secretion [46]. Nevertheless, zebrafish Caspa and Caspb proteins are able to *in vitro* cleave the human GSDMD [47] and zebrafish Gasdermin E [22,48].

### 2.4. Processing of medaka interleukin-1 *in vitro*

**Figure 5.**
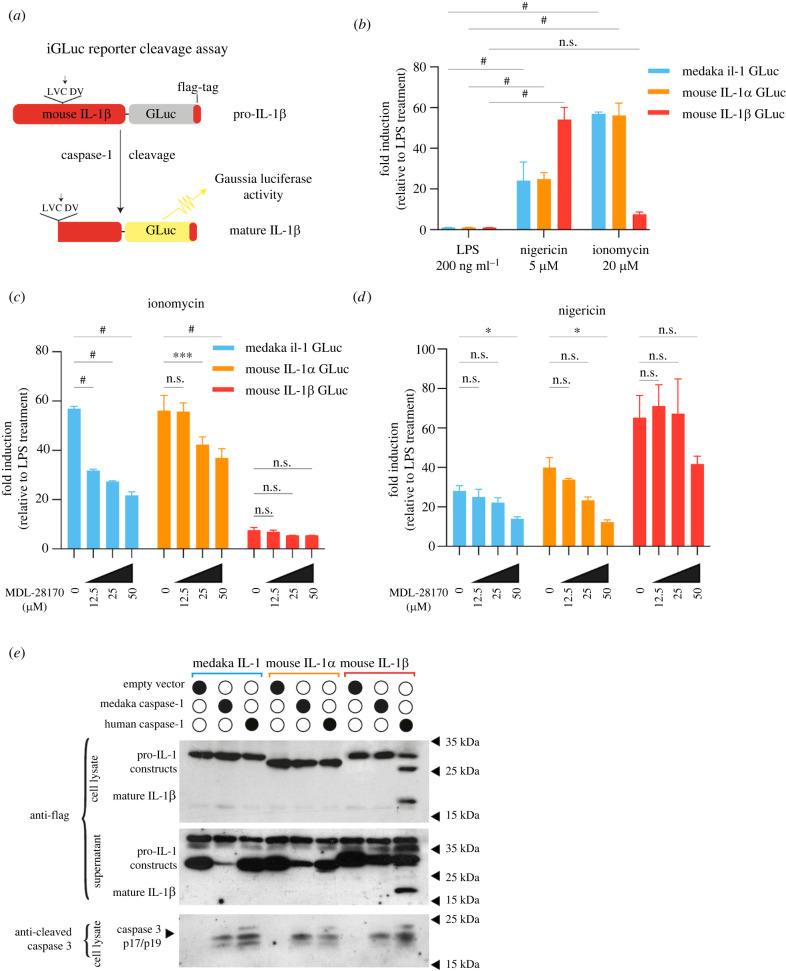
*In vitro* cleavage of mouse IL-1α, IL-1β and medaka Il-1 upon treatment. (*a*) Schematic description of the iGLuc reporter cleavage assay [49]. In this assay, the pro-IL-1β-GLuc fusion protein forms aggregates and is enzymatically inactive. The cleavage of pro-IL-1β-GLuc by Caspase-1 results in monomeric and enzymatically active protein. (*b*) Relative fold-change of luciferase activity upon treatment with different chemical compounds compared to cells treated with only LPS. Mouse J774 macrophages were transfected with pro-IL-1β-GLuc, pro-IL-1α-GLuc and medaka Il-1-GLuc constructs. Data are representative of four independent experiments. (*c*,*d*) Relative fold-change of luciferase activity in transfected J774 cells with different iGLuc constructs and subsequent treatment with ionomycin (*c*) or nigericin (*d*) and MDL-28170. Transfected cells treated with only LPS were used as a control group. WBs confirming the function of the mouse IL-1α and medaka il-1 reporter constructs can be found in the electronic supplementary material, figure S6. (*e*) WB analysis of transfected HEK293T cells with different constructs expressing IL-1 and Caspase-1 cDNAs from the indicated species. The detection of cleaved endogenous Caspase-3 in transfected cells indicates autoactivation of Caspase-1 in 293 T cells, which do not express GSDMD [50–52]. (*b*–*d*) Data depict mean ± s.d. of four independent experiments. Statistical significance was calculated with two-way ANOVA and Dunnet's correction for multiple comparison: * = *p* < 0.05, ** = *p* < 0.01, *** = *p* < 0.001, ^#^ = *p* < 0.0001.

To further test our conclusion that medaka IL-1 is processed in a similar manner as mammalian IL-1α, we compared the processing of medaka IL-1 and mouse IL-1α and IL-1β in a cell-based assay using the pro-IL-1b-Gaussia luciferase (iGLuc) fusion assay [49]. In this assay, pro-IL-1-dependent formation of protein aggregates renders the Gaussia luciferase (GLuc) inactive, and this can be reversed if the cytokine is cleaved, leading to recovery of luciferase activity (figure 5*a*). We transfected mouse J774 macrophages with constructs containing full-length cDNAs of medaka *il1*, mouse *IL1a* or mouse *IL1b* fused with the Gluc reporter. Transfected macrophages were then treated in a similar protocol as in the *in vivo* experiments (figure 4*a*). *In vitro*, LPS alone was not sufficient to induce luciferase activity in macrophages transfected with any of the three IL-1 constructs. However, luciferase became activated when transfected cells were treated with nigericin or ionomycin. Consistent with our previous study [49], luciferase was activated up to 50-fold when cells transfected with mouse IL1β-Gluc were treated with nigericin (figure 5*b*), but not with ionomycin. The effect of nigericin on cleavage of mouse IL-1α and medaka IL-1 was lower. Conversely, ionomycin treatment resulted in a strong luciferase activity in cells transfected with mouse IL-1α-Gluc or medaka IL-1-Gluc constructs (figure 5*b*). The cleavage of medaka IL-1 protein upon ionomycin treatment was further confirmed by WB analysis (electronic supplementary material, figure S6).

Mouse IL-1α has been reported to be cleaved by cysteine proteases. To determine whether this is also true of medaka IL-1, we additionally applied the cysteine protease inhibitor MDL-28170 along with LPS and ionomycin (figure 5*c*) and LPS and nigericin (figure 5*d*). Here, we found a dosage-dependent decrease of luciferase activity for constructs carrying medaka IL-1 or mouse IL-1α after ionomycin and MDL-28170 treatment (figure 5*c*), suggesting that the cleavage of medaka IL-1 also depends on cysteine proteases and further confirming our *in vivo* observations. It has also been reported that pyroptosis induces calpain activation downstream of GSDMD and GSDME pore formation [53,54]. We therefore also tested whether MDL-28170 treatment could inhibit nigericin-induced medaka IL-1 and mouse IL-1α cleavage (figure 5*d*). In our experimental set-up, medaka IL-1 and mouse IL-1α responded similarly at minimal effect. To test whether Caspase-1 can cleave medaka IL-1, we co-transfected 293 T cells with plasmids carrying either medaka or human Caspase-1. We found that human Caspase-1 was only able to cleave mouse IL-1β but not mouse IL-1α or medaka IL-1 (figure 5*e*). Medaka Caspase-1 was not able to cleave any of the tested IL-1 proteins. Together, these results indicate that medaka IL-1 and mouse IL-1α can be processed by cysteine proteases of the calpain or cathepsin family.

### 2.5. Caspase-1 mediated interleukin-1 cleavage in vertebrates

**Figure 6.**
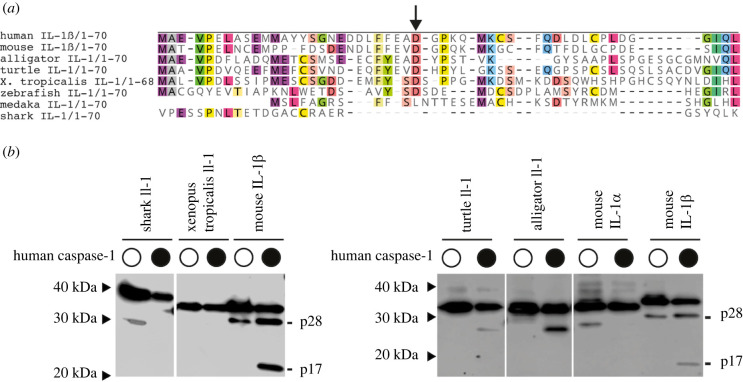
Caspase-1 dependent cleavage of IL-1 in amniotes. (*a*) Alignment of the first 70 amino acids of amniote (human and mouse) and anamniote species, showing a conserved aspartic acid residue (indicated by arrow) between mammalian IL-1β and reptiles IL-1. The accession numbers of genes used are listed in the electronic supplementary material, table S1. (*b*) *In vitro* Caspase-1 assay showing cleavage of turtle and alligator IL-1 by human Caspase-1. Red arrowheads indicate Caspase-1-specific cleavage products.

The processing of IL-1β by Caspase-1 seen in mammals does not appear to occur in medaka IL-1. However, zebrafish IL-1 can be processed by zebrafish Caspase A (also named Casp1) and Caspase B (also named Casp19a) in transfected HEK cells [21–23] and primary zebrafish leucocytes [23]. It is worth nothing that the zebrafish inflammatory Caspases (Caspase A, Caspase b, Caspase 19b and Caspase 23) differ from mammalian Caspase-1 and the Caspase-1 found in other teleost species: while the latter have a Caspase recruitment domain at their N-terminus, zebrafish Caspases A, B and 19b have a Pyrin (PYD) domain instead. Moreover, the mutually dependent activity of Caspase A and B necessary for cleavage of zebrafish IL-1 is not conserved in other vertebrates, and the aspartic acid residues identified by [23] as Caspase-A and Caspase-B-specific cleavage sites are not conserved Caspase-1 cleavage sites in mammals. Therefore, it is likely that zebrafish has independently acquired the ability to be cleaved by caspases. The alignment of IL-1 proteins in vertebrates shows that the N-terminal mammalian IL-1β Caspase-1 cleavage site is conserved in amniotes (figure 6*a*). To determine whether Caspase-1-mediated IL-1 cleavage is characteristic for amniotes, we tested IL-1 proteins from different amniotes (reptiles: alligator and turtle) and anamniotes (fish: shark; amphibian: *Xenopus*). By co-transfecting IL-1 constructs with human Caspase-1, we found that both turtle and alligator IL-1 are cleaved at a site close to the N-terminus, estimated by the product size of around 30 kDa, similar to the intermediate cleavage product of mouse IL-1β (figure 6*b*). By contrast, IL-1 of *Xenopus* and shark could not be cleaved by human Caspase-1. Taken together we show that, first, the expression patterns and protein cleavage of IL-1 in medaka resemble the mammalian IL-1α, and second, the cleavage of IL-1 by Caspase-1 observed has evolved in amniotes. Therefore, a designation of IL-1 in anamniotes as homologue of IL-1β is currently not justified. Additional experimental models will be needed to elucidate the extent to which the cleavage of IL-1 proteins by calpains in anamniotes is necessary for their activity.

## 3. Material and methods

### 3.1. Bioinformatic analysis

Sequences were retrieved using BLASTP searches (http://www.ncbi.nlm.nih.gov/ or http://www.ensembl.org) with default parameters using human and mouse IL-1 proteins. In our phylogenetic tree analysis, we included IL-1 protein sequences from nine mammals, two reptiles, four birds, eight teleosts and one cartilaginous fish. All genes are listed in the electronic supplementary material, table S1. Sequence alignment and phylogenetic trees were done with the Geneious (version 3) software.

### 3.2. Fish

Adult medaka (*Oryzias latipes*) were kept under a 14 h light–10 h dark cycle at 26°C. Embryos were collected and kept in embryonic rearing medium (ERM). Freshly hatched yolksac transgenic larvae were used for most of three experiments. Generation of medaka transgenic reporter lines, and all experimental protocols were performed in accordance with relevant institutional and national guidelines and regulations and were approved by the EMBL Institutional Animal Care and Use Committee (IACUC nos. 2019-03-19ML).

### 3.3. Generation of transgenic fish

To generate transgenic il1:gfp-t2a-*il1*-HA reporter fish, a fragment containing GFP and full-length of medaka il1 cDNA separated by t2a, a short viral sequences, were cloned into a vector containing 6.9 kb upstream region of the *il1* gene (figure 2*a*). The plasmid at 10–25 ng µl^−1^ concentration together with 1 µl *I-SceI* meganuclease and NEB buffer (NewEngland BioLabs) was co-injected into the blastomere at one-cell stage embryos. F0 larvae with GFP signal were selected for breeding.

### 3.4. Immunohistochemistry

Larvae were fixed with 4% paraformaldehyde in 0.1% Tween PBS (1 x PTW). After three washes, larvae were incubated in 30% sucrose/PTW for 24 h followed by 50% tissue freezing medium/30 %Sucrose/PTW for another day. Samples were mounted and sectioned at 20 µM on a cryostat (Leica Biosystems CM2050S). Sections were rehydrated for 20 min with 1 x PTW and blocked with 10% NGS/PTW for 2 h. They were incubated with 1 : 500 mouse-anti-GFP (Sigma) and 1 : 500 Rb-anti-HA antibody (Cell Signaling) in 1% NGS/PTW over night at 4°C. After several washing steps in PTW, sections were stained with anti-Rabbit-Alexa 647 and anti-mouse-Alexa488 in 1% NGS/PTW with 1 : 1000 diluted DAPI for 2 h at 37°C. Slides were washed 3 x 10 min with PTW and then mounted with Vectra shield (Vectra labs). A Zeiss 780 confocal microscope with a 40 x water objective was used for imaging of stained sections.

### 3.5. Flow cytometry

Cells were isolated from spleen, kidney and blood from adult transgenic fish. To avoid blood coagulation, ice cold 0.57 x PBS with 30 mM EDTA was used to collect blood from fish. Cells were disaggregated using a cell strainer (40 µm Nylon, BD Falcon) and collected in FACS buffer (5 mM EDTA, 10 U ml^−1^ Heparin in 1 x PBS). The BD LSR Fortessa Cell Analyzer (BD Biosciences) was used for flow cytometry analysis.

### 3.6. Whole-mount *in situ* hybridization

Whole-mount RNA *in situ* hybridization (WISH) was performed as described previously [55]. Probes used in this study are listed in the electronic supplementary material, table S2.

### 3.7. Wounding assay

The wounding assay was adapted from de Oliveira *et al*. [56]. Briefly, freshly hatched yolksac larvae were anaesthetized in 40 µg ml^−1^ ethyl-m-aminobenzoate methansulfonate (tricaine) in ERM. The caudal fin of larvae was cut using sterile surgical blades. Larvae were then immediately mounted in 1% low-melting agarose containing 40 µg ml^−1^ tricaine and live imaged overnight using confocal microscopes (Zeiss 780 NLO or Leica SP8). The fluorescence intensity over time was calculated using SUM intensity projections, background subtraction; intensity was measured along the line of the cut side or along the rim of the fin in uninjured fins. After background subtraction, the fold-change was calculated from signal intensity at *t*_x_ divided by initial (*t*_0_) fluorescence intensity (*t* = time).

### 3.8. Injection of lipopolysaccharide and bacteria

Anaesthetized larvae were subcutaneously injected with 50 µg ml^−1^ LPS (Sigma) using a glass needle. Anaesthetized adults were injected with PBS containing 20 µg of *Staphylococcus aureus* BioParticles Alexa Fluor 594 conjugate (ThermoFisher) in 50 µl. No fish died as a result of the injection. Adult fish were then kept separately in tanks for 16 h before euthanization and sample preparation for flow cytometry.

### 3.9. *In vivo* interleukin-1 cleavage assay

Freshly hatched yolksac larvae were incubated in 3–4 ml ERM substituted with different combinations of compounds in a six-well plate as shown in figure 4*a*. Larvae were first treated with 50 µg ml^−1^ LPS for 2.5 h followed by 20–50 µm ionomycin (Cayman Chemicals) or 50 µm nigericin (Sigma) for additional 20–60 min. The treatment was terminated when larvae showed clear signs of exposure (immobility). Inhibitors MDL-28170 (Santa Cruz), CA-74 (Cayman Chemicals) and Ac-YVAD-cmk (Sigma) were added directly to the LPS containing medium, and the concentration was kept constant after adding ionomycin into the medium. After treatment, each larva was transferred into a 1.5 ml tube on ice for subsequent protein extraction. Thirty microlitres of protein extraction buffer (10 mM HEPES, 100 mM KCl, 2 mM MgCl_2_, 0,1 mM CaCl_2_, 5 mM EGTA, pH = 8.0, 0.9 mM TritonX, 1 mM NaF, 1 mM Na_3_VO_4_ and proteinase inhibitor) was then added to the tube. Samples were squished using a pestle. The suspension was kept on ice for 20 min and then centrifuged for 20 min 4°C at 10.000 r.p.m. The supernatant was transferred into a new tube and stored at −20°C. To detect proteins, heat-denatured larval suspension were run on a 15% SDS-PAGE and then transferred into a 0.45 µM nitrocellulose membrane by semi-dry electroblotting for 35 min at 13 V. Blots were incubated with 1 : 1000 anti-HA antibody (rabbit, Cell Signaling), 1 : 20 000 anti-GFP (mouse, Sigma) and 1:20 000 anti-actin (rabbit, Sigma).

### 3.10. *In vitro* interleukin-1 cleavage assay

For lentiviral overexpression in J774 macrophages, murine IL1a-GLuc, murine IL1b Gluc and medaka IL1 were subcloned into third-generation lentivector pLenti6-EF1alpha-IRES-EGFP (a derivative of Invitrogen pLenti6, kindly provided by Jonas Doerr, Institute of Reconstructive Neurobiology, University of Bonn) via SalI/NotI fusion. Lentiviruses were generated using calcium-phosphate transfection of HEK293T, and J774 macrophages were spin transduced, as described in [57] and sorted for GFP expression. For inflammasome experiments, the luciferase signal was measured directly from the supernatant after the addition of the Gaussia luciferase substrate coelenterazine as performed in [49].

For transient transfection, murine IL1a-GLuc, murine IL1b Gluc and medaka IL1 were subcloned into the mammalian expression vector pEFBOS containing a C-term FLAG-tag via *XhoI*/*BamHI* fusion. Medaka caspase-1 and human caspase-1 were subcloned into pLenti6-EF1alpha-IRES-EGFP. HEK293T cells were transfected with the indicated plasmids using TransIT-LT1 (Mirus Bio). Cells were lysed with SDS-sample buffer 24 h after transfection and prepared for immunoblotting. To detect proteins, heat-denatured samples were run on a 12% SDS-PAGE and then transferred into a 0.2 µM nitrocellulose membrane using wet transfer (50 min, 100 V). Blots were incubated with 1 : 1000 Monoclonal ANTI-FLAG^®^ M2- HRP antibody (mouse, Sigma) or 1 : 1000 anti-cleaved Caspase-3 #9661 (rabbit, Cell Signaling Technology), followed by 1 : 3000 goat anti-rabbit HRP 1706515 (BioRad).

### 3.11. Statistical analysis

Wilcoxon–Mann–Whitney test was used to calculate significant differences where indicated. A *p*-value less than 0.05 was considered statistically significant. The numbers of biological samples (*N*) for experiments are indicated in each figure. Data in bar graphs are shown as an absolute number with means ± s.d. noted. All data were analysed in GraphPad Prism software (version 9).

## Data Availability

This article has no additional data.
